# Hypoxia-associated genes predicting future risk of myocardial infarction: a GEO database-based study

**DOI:** 10.3389/fcvm.2023.1068782

**Published:** 2023-07-03

**Authors:** Shaohua Li, Junwen Zhang, Jingwei Ni, Jiumei Cao

**Affiliations:** ^1^ Department of Cardiology, Shandong Provincial Hospital Affiliated to Shandong First Medical University, Jinan, China; ^2^ Department of Cardiothoracic Surgery, Xinhua Hospital Affiliated to Shanghai Jiaotong University School of Medicine, Shanghai, China; ^3^ Department of Cardiovascular Medicine, Ruijin Hospital, Shanghai Jiao Tong University School of Medicine, Shanghai, China; ^4^ Department of Geriatrics, Ruijin Hospital, Shanghai Jiaotong University School of Medicine, Shanghai, China

**Keywords:** coronary artery disease, myocardial infarction, GEO, WGCNA, GWAS, CSTF2F, unstable angina (UA)

## Abstract

**Background:**

Patients with unstable angina (UA) are prone to myocardial infarction (MI) after an attack, yet the altered molecular expression profile therein remains unclear. The current work aims to identify the characteristic hypoxia-related genes associated with UA/MI and to develop a predictive model of hypoxia-related genes for the progression of UA to MI.

**Methods and results:**

Gene expression profiles were obtained from the GEO database. Then, differential expression analysis and the WGCNA method were performed to select characteristic genes related to hypoxia. Subsequently, all 10 hypoxia-related genes were screened using the Lasso regression model and a classification model was established. The area under the ROC curve of 1 shows its excellent classification performance and is confirmed on the validation set. In parallel, we construct a nomogram based on these genes, showing the risk of MI in patients with UA. Patients with UA and MI had their immunological status determined using CIBERSORT. These 10 genes were primarily linked to B cells and some inflammatory cells, according to correlation analysis.

**Conclusion:**

Overall, GWAS identified that the CSTF2F UA/MI risk gene promotes atherosclerosis, which provides the basis for the design of innovative cardiovascular drugs by targeting CSTF2F.

## Introduction

Coronary Heart Disease (CHD) conteins myocardial infarction (MI), angina pectoris, and coronary arteriosclerosis (1, 2). The incidence and mortality of CHD in developing countries, including China, are increasing year by year (3, 4). MI is one of the most serious manifestations of coronary artery disease (CAD) and the leading cause of death from non-infectious diseases worldwide (5). Atherosclerosis has been recognized as the main underlying mechanism of coronary heart disease, ultimately leading to acute coronary syndrome (ACS). ACS includes a clinical spectrum ranging from unstable angina (UA) to acute MI (6). Currently, the treatment of UA is mainly conservative drug treatment, such as anti-platelet aggregation, coronary artery dilation, lipid reduction, etc., to prevent the progress of UA, thus reducing the risk of MI (7, 8). However, the molecular mechanism from UA to MI is still poorly understood.

Ischemic/hypoxic injury is a common pathological basis for CHD, atherosclerosis, and other cardiovascular diseases. Coronary atherosclerosis can lead to narrowing of the vessel lumen, and angina pectoris caused by this rapid, transient myocardial ischemia and hypoxia is a common symptom in the chest (9), and myocardium with underdeveloped collateral circulation is susceptible to ischemic necrosis, leading to MI (10, 11). Coronary atherosclerosis is the main pathological basis of coronary heart disease, of which atherosclerotic plaque consists mainly of cholesterol and cholesteryl esters, which are highly brittle and prone to rupture or dislodge and flow with the blood, blocking small vessels or capillaries, forming thrombi, and aggravating MI (12, 13). Atherosclerotic plaque rupture has been identified as a major cause of ACS (14). Nevertheless, non-atherosclerotic factors have been identified to cause ACS (15). Therefore, further comparison of alterations in hypoxia genes in UA progression to MI must be done with association analysis of atherosclerosis-related protective factors and suppressors to determine the potential role of these genes in atherosclerosis-mediated or non-atherosclerosis-mediated ACS. Thus hypoxia-related genes may be potential targets for CAD disease progression and treatment.

The clinical occurrence of MI is often a sudden symptom. Early prevention and intervention are difficult to achieve. However, early MI has lesions such as coronary arteries, blood composition, and hormone levels. Therefore, MI can be prevented at an early stage by molecular level changes as markers (16). Recent studies in transcriptomics have been continuously applied in clinical practice, especially in routine diagnostic applications. Microarray data and RNAseq data are widely used for disease diagnosis. Numerous studies have confirmed the abnormal expression levels of some disease-related genes in the early stages of MI. Zhang et al. have then been in the process of identifying the RNA signature of coronary heart disease from the combined expression profile of lncRNA and Mrna (17). A study folded into diagnostic features byMI-related differentially expressed genes (18). We, therefore, sought to identify hypoxia-associated genes for molecular characterization from UA to MI while trying to find their association with atherosclerosis in anticipation of finding available therapeutic targets.

## Materials and method

### Data collection and organization

We started from GPL571, GPL6106-based platform (GSE48060, GSE61144, GSE29111, GSE97320, GSE34781) Gene Expression Omnibus (GEO) database (https://www.ncbi.nlm.nih.gov/geo/) to obtain a total of 93 samples (18 UA cases and 75 IM cases) for training set analysis. A total of 24 samples (8 UA cases and 16 MI cases) were also obtained from the GPL6884-based platform (GSE60993) for test set validation. The microarray data were quantile normalized to obtain standardization (19). The microarray probes were converted to 12,285 Ensembl gene IDs using the biomaRt software package. According to the standardized sample network connectivity *Z*-score < −2, the samples defined as outliers are removed (20). The batch effects of the expression profiles are processed using the operational function of the SVA package in R (21). All data processing results are shown in Supplementary Figure S1. Furthermore, total of 25 normal samples from GSE34781, GSE48060, and GSE97320 were used to compare the expression levels of the core genes in the normal, UA and MI groups. Detailed sample sizes for the GEO dataset are provided in Supplementary Table S1 and clinical information is provided in Supplementary Table S2.

### Identification of genes related to hypoxia

In the training data, UA was compared with MI to identify genes with significant expression differences. We use the “limma” package R to extract differentially expressed genes (DEGs). After correction based on the false discovery rate, the cutoff value of the *P* value was set to 0.05 to obtain 3,569 differential genes considered as disease progression genes. Subsequently, we used “ssGSEA” to quantify the Hallmark gene set obtained from MSigDB (gsea-msigdb.org/gsea/msigdb/). Total of 4,017 differential genes related to hypoxic were identified by differential analysis. In parallel, we built a weighted gene co-expression network analysis (WGCNA) and identified hypoxia-associated modules by package “WGCNA” (22). Interactions between unique genes with hypoxic ssGSEA (23) scores were quantified by gene significance (GS), and correlations between gene expression profiles and module signature genes were indicated by module membership (MM). At a threshold of GS *p* < 0.05 threshold, 630 candidate genes from the “sapphire-colored module” were selected. Differential genes were analyzed by “ClusterProfiler” R (24) package to perform GO, KEGG enrichment.

### Classification model construction and validation

The ten genes with the highest coefficients in the LASSO regression model were selected for the construction of the model by selecting 630 modular genes for the R package “glmet” (25). The hypoxic risk score was calculated using the corresponding coefficients of the selected features. The formula was established as follows.

$$\mathrm{Risk}\;\mathrm{score}=\overset{n}{\underset{i=1}{\sum }}\mathrm{Coe}f_{i}*x_{i}$$

where $\mathrm{Coe}f_{i}$ denotes the coefficient and $x_{i}$ denotes the expression value of each mRNA. Patients were divided into low- and high-risk groups according to the median value of risk value, and PCA downscaling analysis showed its distribution pattern of patients with different risks. ROC curves were used to assess model accuracy. A validation set was used to verify the above results.

### Estimation of immune cell infiltration

The CIBERSORT (26) algorithm was used to translate normalized gene expression values into the composition of immune cells in complex samples. CIBERSORT generates an empirical *P*-value for the inverse convolution results using Monte Carlo sampling, which is used to compare the statistical significance of the inverse convolution results on all subsets of cells. The training set of 93 samples was included in the CIBERSORT analysis. The entire sample was included in the follow-up analysis (all *P*-values less than 0.05). The specific results are given in Supplementary Table S3. The ratio of immune cells in significantly enriched UA and MI samples was obtained and reported as a bar graph. We used the R package “vioplot” to compare the levels of each immune cell type. Spearman's rank correlation analysis was performed to explore the correlation between infiltrating immune cell subsets and signature genes. The R package “ggplot2” is used to visualize plots.

### eQTL mapping in GTEx

SNP loci associated with UA/MI disease were obtained from the GWAS catalog database. The public eQTL database was queried in the Genotype-Tissue Expression (GTEx) project (Release V8) to determine the association between lead UA/MI GWAS variants and CSTF2T gene expression. GTEx tissue-specific eQTL were also identified for the GWAS variants using 48 different GTEx tissues sorted by NES value and eQTL *p*-value.

### Western blot

Whole blood samples were obtained from 3 cases of UA and 3 cases of MI patients, and 3 cases of healthy volunteers in Ruijin Hospital, and kept in EDTA evacuated tubes. Subsequently, peripheral blood monocytes (PBMCs) were isolated using Ficoll-Hypaque density gradient centrifugation (TBD Science, Tianjin, China). Total protein was isolated from the isolated PBMCs in ice-cold lysis buffer using a high-intensity ultrasomic processor, according to the manufacturer's instructions. The remaining debris were removed by centrifugation, and the protein was precipitated with cold 15% Trichloroacetic for 2 h at −20 °C. After centrifugation at 20,000 × g at 4 °C for 10 min, the supernatant was discarded. The protein was dissolved in buffer, and the protein concentration was determined with a 2-D Quant kit according to the manufacturer's instructions. Equal amount of protein samples were subjected to 10% SDS-PAGE and transferred to PVDF membranes. After blocking at 37 °C for 2 h, the membranes were probed with primary antibodies at 4 °C overnight. After washing with TBST, the blots were incubated with a secondary antibody at 37 °C for 1 h. Immunoreactivity was visualized by enhanced chemiluminescence. The intensity of the bands was normalized to that of GAPDH. The statistical significance of changes in protein expression observed by Western blotting was assessed with a two-tailed t-test using GrapghPad Prism 8.0.2. The data are expressed as the mean ± standard deviation, and statistical significance was considered to be indicated by a *p*-value < 0.05.

### Statistical analysis

All statistical analyses were performed using R software (version 4.0.3). The Wilcoxon test was used to compare the differences between the two groups. The Spearman's rank correlation analysis was used to explore the correlation between model genes and immune cells. Values of *p* < 0.05 were considered statistically significant.

## Result

### Hypoxia-related gene basis of coronary heart disease

**Figure 1:**
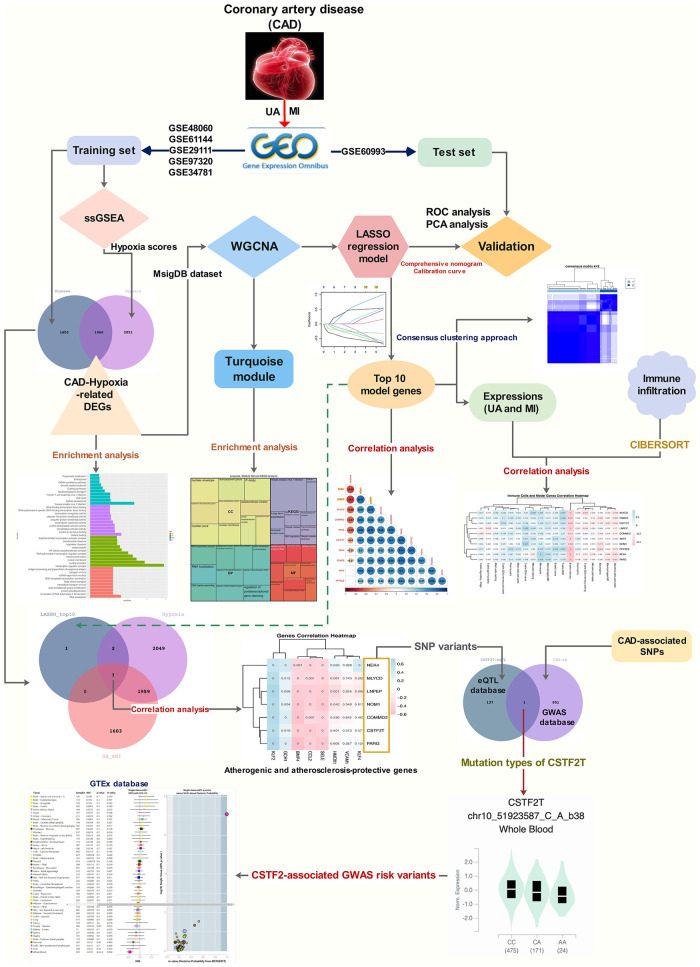
The workflow of the integrative bioinformatics analyses.

**Figure 2:**
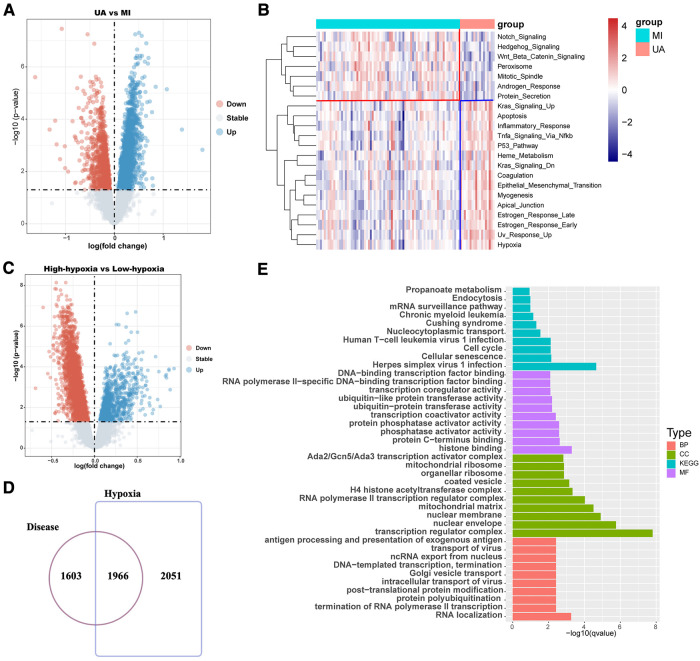
Difference analysis showed that hypoxia was associated with the development of coronary heart disease. (**A**) The volcano map shows the different results between UA and MI. (**B**) Hypoxia ssGSEA scores were estimated in the UA and MI cohort by performing ssGSEA with a hallmark gene set. (**C**) The volcano map shows the different results between high-hypoxia scores and low-hypoxia scores. (**D**) Venn diagram shows that disease differential genes overlap with hypoxia differential genes. (**E**) Enrichment analysis of disease-differential genes. UA: unstable angina; MI myocardial infarction; BP: Biological Process; CC: Cellular Component; MF: Molecular Function.

The workflow is shown in Figure 1. Many patients with MI have rapidly progressing disease due to UA (27). We divided the patients into two groups based on two different clinical phenotypes, including 18 UA patients and 75 MI samples. Using the limma algorithm, we finally screened 3,569 DEGs between UA and MI groups based on a corrected *P* value < 0.05 (Figure 2A). Atherosclerosis exacerbated by ischemia/hypoxia eventually led to stable and UA and MI, and the differently enrichment of pathways between the two groups is shown in the heatmap, we then calculated the hypoxia scores of UA and MI patients according to ssGSEA (Figure 2B), and the difference analysis between high-hypoxia and low-hypoxia groups obtained 4,017 DEGs displayed in the volcano plot (Figure 2C). The 1966 intersecting genes were demonstrated by VENN plots (Figure 2D). Since our results indeed show that hypoxia is a prominent molecular feature of UA, these intersecting genes are regarded to be the molecular basis of hypoxia-related genes in UA to MI disease progression. We then performed GO and KEGG on these genes, which were highly enriched in propanoate metabolism, chronic myeloid leukemia, cell cycle, mitochondrial ribosome, H4 histone acetyltransferase complex, and cellular senescence pathways (Figure 2E), which are all related to oxygen metabolism/hypoxia (28–33).

### Identification of hypoxia-related genes by weighted gene coexpression network analysis

**Figure 3:**
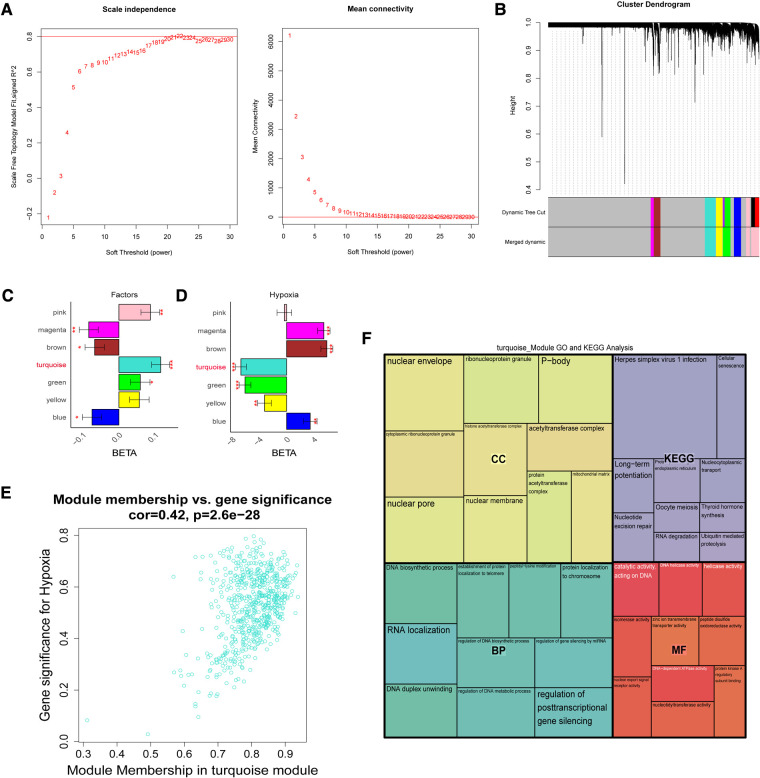
The weight gene co-expression network analysis showed that hypoxia was associated with the development of coronary heart disease. (**A**) Soft threshold selection process. (**B**) Cluster dendrogram. Each color represents one specific co-expression module. In the colored rows below the dendrogram, the two colored rows represent the original modules and the merged modules. (**C-D**) The differential expression of eigengenes in UA and MI, high hypoxia and low hypoxia (FDR corrected *P < 0.05, **P < 0.01, ***P < 0.001), respectively. (**E)** Turquoise gene significance and membership in hypoxia network. (**F**) Gene ontology and KEGG pathways enrichment in turquoise modules.

To further screen for reliable genes associated with hypoxia. Based on the ssGSEA method and cancer markers from the MsigDB dataset, the hypoxia ssGSEA Zscore was calculated for the training set of patients in our dataset, and candidates associated with hypoxia were screened by WGCNA. The optimal soft threshold was determined to be 21 (Figure 3A), Dynamic Tree Cut was set to 0.2, and 7 modules were created (Figure 3B), with the turquoise-colored module having the highest correlation with UA/MI (Figure 3C), while we compared the correlation of hypoxia scores with the modules. The turquoise module possessed the highest correlation with the hypoxic phenotype (Figure 3D). From the turquoise module, 630 promising candidates were identified (Figure 3E). We performed GO, and KEGG analysis on these genes, and not surprisingly these genes were associated with Thyroid hormone synthesis, Ubiquitin mediated proteolysis, Nucleotide excision repair, Cellular senescence, and histone acetyltransferase complex (Figure 3F), which are related to oxygen metabolism/hypoxia (34–38).

### Classification model construction and validation

**Figure 4:**
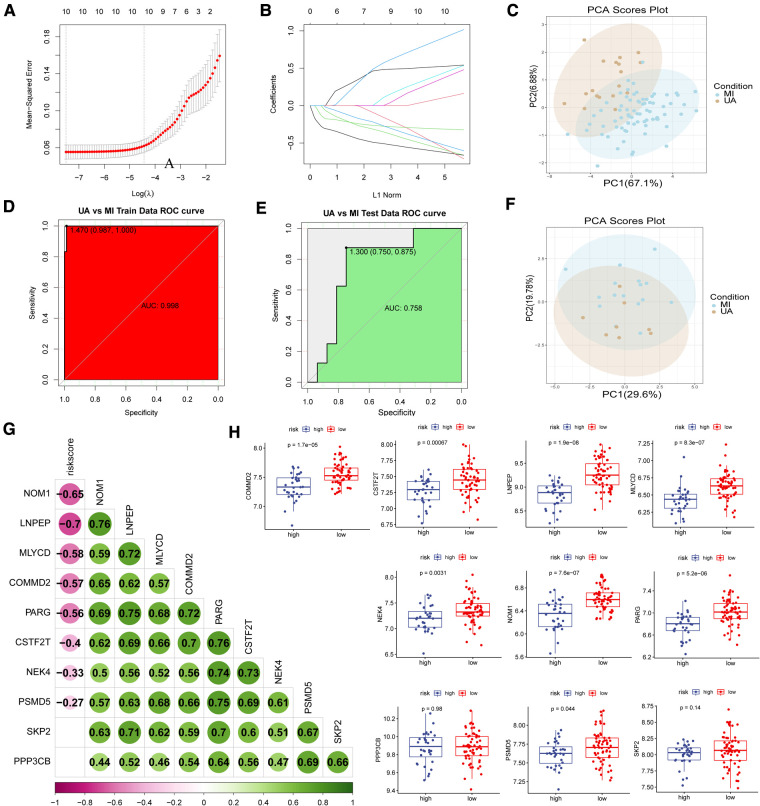
Construction of disease classification model. (**A-B**) The lasso regression top 10 genes in training data. (**C**) PCA analysis in train data and (**F**) test data. ROC analysis in train (**D**) data and (**E**) test data. (**G**) Correlation analysis of risk scores and model genes. (**H**) Model gene expression in high and low-risk groups.

Based on the lasso algorithm, the top 10 genes with the highest coefficients (COMMD2, CSTF2T, LNPEP, MLYCD, NEK4, NOM1, PARG, PPP3CB, PSMD5, and SKP2) were selected (Figures 4A–B). PCA analysis showed that the samples were divided into two clusters (Figure 4C). The area under the ROC curve in the training set was 0.998 (Figure 4D), demonstrating its excellent classification ability, and these results were confirmed in our test set (Figures 4E–F). We found that age was positively associated with risk score in GSE29111 (R = 0.29; *P* < 0.05), but not gender (Supplementary Figure S2). We also analyzed the classification ability of individual genes (Supplementary Figure S3). Furthermore, we compared Pearson's correlations between these genes and risk scores, and most of them showed high correlations, with NOM1, and LNPEP showing a strong correlation (|R|>0.6, *p* < 0.05) (Figure 4G). The high expression of most hypoxia-related model genes represents a low risk of MI incidence in these patients (*p* < 0.05) (Figure 4H).

### Construction of the nomogram model

**Figure 5:**
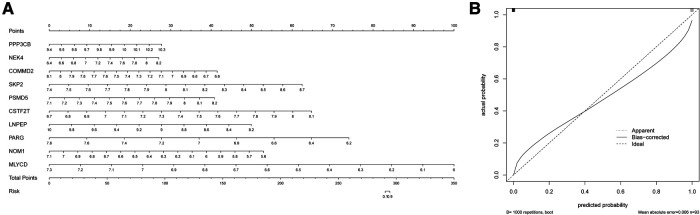
Construction of the nomogram. (**A**) Comprehensive nomogram of 10 risk genes. (**B**) Calibration curve.

The nomogram is a simple, personalized visualization tool that has been widely used in disease diagnosis and prognosis (39). We used the “rms” package in R to construct a nomogram model based on 10 hypoxia-related genes to predict the prevalence of MI in UA (Figure 5A). The column line plot c-index value is 1. The calibration curve shows that the predicted results based on the column line graph are in good agreement with the actual prognostic results (Figure 5B).

### Generation of hypoxia gene signatures

**Figure 6:**
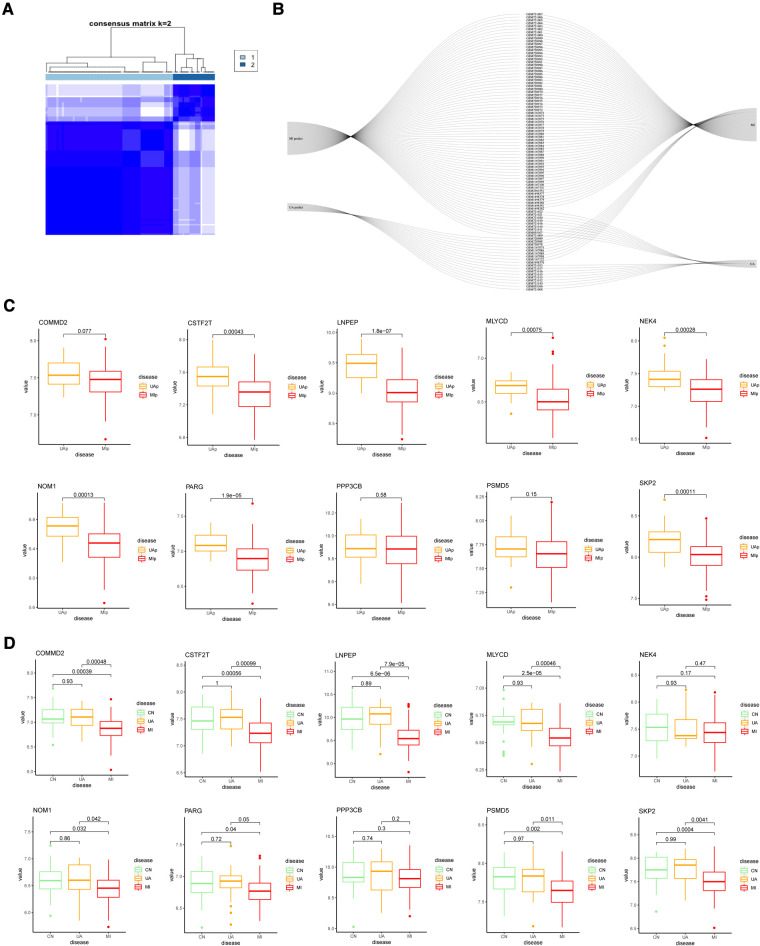
Consensus clustering of 10 important hypoxia-related genes in UA/MI. (**A**) Consensus matrices for k = 2. (**B**) Sankey diagram showing the relationship between UA, MI, and UAp, MIp modes. (**C**) Expression of 10 hypoxia-related genes in UAp, MIp modes. (**D**) Expression of 10 hypoxia-related genes among CN, UA, and MI groups.

To further validate the precision of the hypoxia pattern, patients with UA/MI were classified into different genomic subtypes using a consensus clustering approach based on 10 hypoxia-associated model genes. We found the existence of two different hypoxia gene patterns (gene cluster 1 and gene cluster 2) (Figure 6A). The Sankey diagrams visualized the relationship between predicted UA and MI patients based on hypoxia-related genes and actual patients (Figure 6B). In addition, we compared the expression of these hypoxic genes between predicted UA and MI patients (Figure 6C), and the results were similar to those in the risk model, except for COMMD2, PPP3CB, and PSMB5, which were all downregulated in MI patients (*p* < 0.05). This again validates the accuracy of our grouping by the consensus clustering method. To further explore the relationship between these ten genes and the progression of UA/MI disease. We found that the mRNA expression of COMMD2, CSTF2T, LNPEP, MLYCD, NOM1, PARG, PSMD5, and SKP2 was not significant between the normal and UA groups, but their expressions were different between the UA and MI groups (*p* < 0.05) (Figure 6D). This suggests that these genes change as the disease progresses.

### Correlation of gene expression and immune cell infiltration

**Figure 7:**
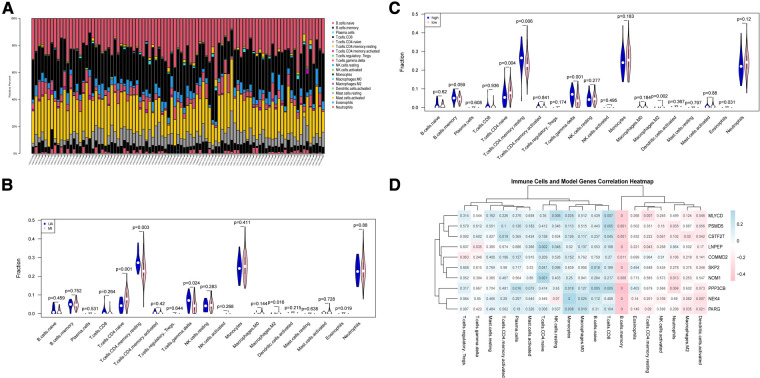
Immune cell subsets analysis in UA/MI. (**A**) The proportion of immune cell subsets in UA/MI samples. (**B**) Differences in analysis of immune cell infiltration between UA and MI patients. (**C**) Differences in analysis of immune cell infiltration between high- and low-risk groups. (**D**) 10 hypoxia-related genes associated immune cells. NES: normalized enrichment score.

Atherosclerosis is a chronic inflammatory disease. It is characterized by complex immune interactions between resident vascular cells and specialized immune cells (40). For example, type 2 macrophages are also associated with repair and reconstruction at sites of inflammation (41). Therefore, we sought to find differences in the immune environment between UA and MI. The CIBERSORT algorithm was performed to assess the ratio of immune cell subsets in the training set. Total of 22 immune cell subpopulations are shown in Figure 7A. We found that the relative percentage of T.cells.CD4.naive was higher in the MI group compared to UA, while the relative percentages of CD4 memory resting, T cells gamma delta and relative percentages were significantly less (*p* < 0.05) (Figure 7B). In addition, we compared high and low-risk groups with immune cells and obtained the same results as disease groups (Figure 7C). Furthermore, we performed a correlation analysis of risk scores and immune cells. As shown in Supplementary Figure S4, among the above immune cells with differences, except for macrophage M2, which does not correlate with a risk score, the remaining 4 immune cells include T cells CD4 naive, T cells CD4 memory resting, T cells gamma delta and Eosinophils were all correlated with risk score (|R|>0.2; *P* < 0.05). We then did a correlation analysis of immune cells and 10 hypoxia-related genes (Figure 7D). An interesting phenomenon caught our attention, all hypoxia genes showed a negative correlation with memory B cells (|R|>0.2; *p* < 0.05), and we found that macrophage M2 showed a negative correlation with CSTF2F and PARG (*p* < 0.05), and CD8+ T cells MLYCD, CSTF2F, PSMD5 and PPP3CB showed a positive correlation (*p* < 0.05). Evidence for an important role of B lymphocytes in human CVD is limited. In patients with acute myocardial infarction (MI), high levels of the B-cell specific cytokines and B-cell activating factor, predict increased risk of death and recurrent MI (42). B cells are important in immune homeostasis.

These findings indicated that the hypoxia-related model composed of 10 genes may affect the infiltration and proportion of immune cells by driving the hypoxic state of immune cells, thereby regulating the immune homeostasis of UA/MI patients.

### UA/Mi-associated risk variants in the CSTF2T locus are associated with reduced CSTF2T gene expression

**Figure 8:**
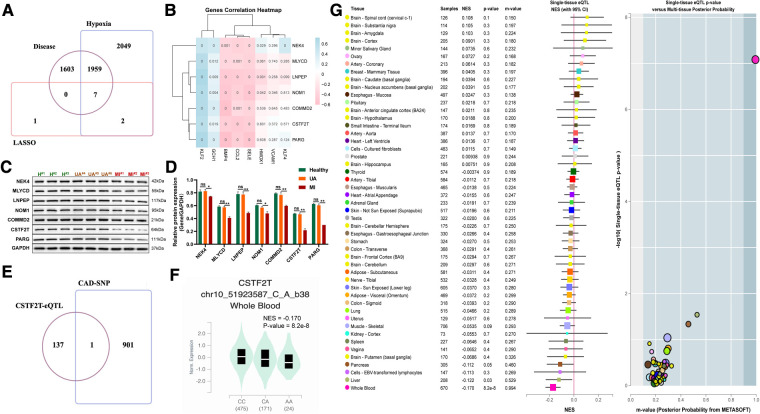
Correlation between mutations at disease-associated hypoxia gene loci and their expression. (**A**) Venn diagram among model genes, hypoxia differential genes, and differential genes between UA and MI. (**B**) Association of seven disease-related hypoxia genes with atherosclerosis. (**C**) Western blot validation for the relative protein expression levels of seven disease-related hypoxia genes (NEK4, MLYCD, LNPEP, NOM1, COMMD2, CSTF2T, PARG) among healthy volunteers, UA and MI patients. (**D**) Statistical results of protein bands in C. (**E**) VENN plot of CAD GWAS and eQTL analysis results of seven disease-related hypoxia genes. (**F**) Boxplots showing expression quantitative trait locus data from the GTEx dataset for a genome-wide association study of the SNP (rs2879627) association. (**G**) Normalized effect sizes for single-tissue expression quantitative trait loci normalized effect sizes for rs2879627 are shown for 48 different tissues in GTEx.

To further identify key genes driving UA/MI development, we intersected the genes in the model with disease risk genes and identified 7 genes that were strongly associated with disease development (Figure 8A). Notably, these 7 genes were negatively associated with the expression of atherogenic genes (CCL2, BMP4, and SELE) and positively associated with multiple atherosclerosis-protective genes (KLF2 and GCH1) (Figure 8B). Further western blot validation was performed, and the results indicated that the expression levels of seven genes (NEK4, MLYCD, LNPEP, NOM1, COMMD2, CSTF2T, PARG) related to disease and hypoxia were significantly reduced in MI patients compared with healthy volunteers, but their expression were not significant between the healthy and UA samples (Figures 8C–D). The 902 SNPs associated with UA/MI were identified by mining the GWAS catalog database to take intersections with these 7 gene SNP loci. We found that only the rs2879627 variant in the CSTF2T locus was associated with UA/MI occurrence (Figure 8E) and determined the mutation type of CSTF2T expression (Figure 8F). By mining genotype and transcriptome data in the Genotype-Tissue Expression (GTEx) database, we mapped the main GWAS risk variants associated with CSTF2 expression (rs2879627 locus) in human tissues and determined that the rs2879627 mutation was associated with CSTF2 expression in whole blood (NES < −0.1; *p* < 0.05) (Figure 8G).

### Discussion

One of the major diseases that endanger human health is CAD. The most common reason for death in people with cardiovascular disease is MI, in particular (43). Therefore, rapid implementation of successful treatment requires an early, quick, and correct diagnosis and assessment of MI (44). We report for the first time that blood markers of MI from UA can be identified and a MI warning issued.

ACS is the most serious medical emergency caused by myocardial ischemia/hypoxia following coronary atherosclerotic plaque formation, with clinical manifestations of UA and acute MI (45). Hypoxia is an inevitable manifestation of atherosclerosis due to vessel wall thickening (46). Given this, we investigated the potential value of hypoxic gene signatures in this study. The hypoxic status of UA/MI was assessed by using ssGSEA and WGCNA methods, respectively, to select hypoxia-associated genes. Our discovery that differential genes based on hypoxia scores were largely the same as those of UA and MI, especially the conversion process to MI, demonstrates that hypoxia is intimately related to UA/MI. Regarding these frequent genes, GO and KEGG analyses revealed that they were enriched in propanoate metabolism, chronic myeloid leukemia, cell cycle, mitochondrial ribosome, H4 histone acetyltransferase complex, and cellular senescence pathways, which are all related to oxygen metabolism/hypoxia (28–33). These hypoxia-related pathways and functions were strengthened. In UA/MI patients, this guarantees the exclusivity and specificity of our identified gene signature. Then, to reflect the categorical gene profile of patients going from UA to MI, our WGCNA technique identified the most relevant modules for hypoxic characteristics and chose the top 10 genes with the highest weights by the LASSO regression model. The categorization algorithm was applied to forecast the risk that UA patients would advance to MI. PCA analysis showed that the classification model had different distribution patterns. The area under the ROC curve for the training set was 1 and the area under the ROC curve for the test set was 0.758, indicating that the classification model has satisfactory performance. In addition, we constructed a nomogram model based on 10 candidate hypoxia-related genes and the calibration curves showed that the decisions based on the nomogram model were accurate. We found that most of the genes in the model were significantly associated with risk scores and high and low-risk groupings in our study, and two disease patterns (cluster1 and cluster2) were identified based on 10 significant hypoxia-related genes using consensus clustering methods. These two patterns were reflected as UA prediction (UAp) and MI prediction (MIp). The Sankey diagram shows that the UAp and MIp determined based on the consensus clustering of these 10 genes are highly consistent with the actual UA, and MI patients. And the UAp and MIp groupings had the same expression levels of the 10 hypoxic genes as those of high- and low-risk patients. Thus, these results confirm the reliability of the 10 hypoxia-gene signatures for differentiating UA and MI patients.

Inflammation is a key factor in the development and progression of atherosclerosis (47). Myocardial infarction has always been considered an inflammatory disease, and the occurrence, development, and prognosis of MI are closely related to the overexpression of inflammatory cytokines (48, 49). Therefore, we tried to find the consensus and uniqueness of the immune signature of UA and MI. The percentage of immune cells in patients with UA and MI was calculated using the CIBERSORT algorithm. T cells CD4.naive was found to be higher in the MI group compared to UA, while the relative percentages of CD4 memory resting, T cell gamma delta, and macrophage M2 were significantly lower in the MI group. Recent studies have shown that the infiltrating macrophages polarization affects the expression of pro- and anti-inflammatory cytokines in the epicardial adipose tissue from UA/MI patients and that the ratio of M1/M2 macrophages is positively correlated with the severity of CAD (50). This is consistent with the phenomenon of reduced macrophage M2 in patients with MI found in our study. In addition, we found that all model genes were highly correlated with B cells. Although B cells were not previously reported to be the predominant immune cell type found in atherosclerotic lesions (51). However, they are abundant in perivascular adipose tissue, which, in addition to the spleen and bone marrow, serves as a niche for immunoglobulin (Ig) production (52). Cell production of cytokines and Ig by B-cell production of cytokines and Ig at these sites is thought to be an important regulator of inflammation in atherosclerotic lesion formation (53). B-cell depletion reduced the development of atherosclerosis in mice (53). At the same time, we learned that the hypoxia and hypoxia-inducible factor (HIF) signaling pathways are critical for B cell development and function, such as survival, proliferation, and cytokine production of B cells (54), and that improper regulation of this B cell can lead to a variety of diseases, including atherosclerosis. In patients with acute MI, high levels of the B-cell specific cytokines and B-cell activating factor, predict increased risk of death and recurrent MI (42). B cells are important in immune homeostasis. Therefore, our findings indicated that the hypoxia-related model composed of 10 hypoxia-associated genes may affect the infiltration and proportion of immune cells by driving the hypoxic state of B-cell activity, thereby regulating the immune homeostasis of UA/MI patients.

The pathological underpinning for CAD is atherosclerosis, and by crossing model genes with disease-associated hypoxia genes, we were able to identify 7 shared genes. These 7 genes were shown to be positively correlated with several atherosclerosis preventive genes and negatively correlated with the expression of atherogenic genes (CCL2, BMP4, and SELE), according to correlation analysis (KLF2 and GCH1). A large-scale genome-wide association study (GWAS) is currently being conducted has found hundreds of potential loci linked to the risk of UA and MI risk (55). However, the genetic mechanisms and causative genes of the UA/MI locus have not been fully resolved, so we sought to find correlations between UA/MI causative genes and our findings. We integrated GWAS and whole-tissue Expression Quantitative Trait Loci (eQTL) analysis to explore hypoxia-related gene expression and its disease SNP association.

### Strengths and limitations

Strengths of this study focused on that we found that CAD GWAS risk variants at the CSTF2T locus were closely associated with increased CSTF2T expression in whole blood tissue, suggesting that CSTF2T may promote atherosclerosis, which may be a future direction for the treatment of atherosclerosis. However, the present study had the following limitations. First, the study was a single-centered study; therefore, the results cannot be generalized to other populations with varying demographics. Second, the less number of patients and further research is required to determine whether CSTF2T could be a better indicator for predicting the incidence of MI in UA patients. Despite these meaningful findings, we had to face some limitations. Our study is based on publicly available data. Therefore, more external cohorts are needed to validate our findings. Again, due to the difficulty of obtaining clinical information, we were unable to describe the association of risk scores with more clinical features including reinfarction, the presence of atherosclerosis.

## Conclusion

In conclusion, the current study provides insight into hypoxia-related genes and establishes 10 hypoxia-related gene signatures to predict the incidence of MI in patients with UA. At the same time, a nomogram was constructed based on these genes, showing the risk of MI in patients with UA. Immune subsets analysis showed that these ten genes were mainly associated with B cells and some inflammatory cells. Furthermore, the UA/MI risk gene CSTF2F identified by GWAS promotes atherosclerosis, which provides a rationale for designing innovative cardiovascular drugs by targeting CSTF2F.

## Data Availability

The raw data supporting the conclusions of this article will be made available by the authors, without undue reservation.
