# Supplementary material for: Hypoxia-associated genes predicting future risk of myocardial infarction: a GEO database-based study
Source: Front Cardiovasc Med. 2023 Jul 3;10:1068782. doi: 10.3389/fcvm.2023.1068782 (PMC10351911; doi:10.3389/fcvm.2023.1068782)
Supplement: Supplementary file 1 [file Table1.docx]

**Table S1：GEO datasets**.

|  | **Study** | **Platform** | **Tissue** | **Cases** | **Total** |
| --- | --- | --- | --- | --- | --- |
| Training Set | GSE48060 | GPL570 | Peripheral blood | MI (n = 31) | 93 samples (18 UA cases and 75 MI cases) |
|  | GSE61144 | GPL6106 | Peripheral blood | MI (n = 7) |  |
|  | GSE29111 | GPL570 | whole blood | UA (n = 16) MI (n = 36) |  |
|  | GSE97320 | GPL570 | peripheral blood | MI (n = 1) |  |
|  | GSE34781 | GPL570 | white blood cell of venous blood | UA (n = 2) |  |
| Testing Set | GSE60993 | GPL6884 | Peripheral blood | UA (n = 8) MI (n = 16) | 24 samples (8 UA cases and 16 MI cases) |
| Normal tissue | GSE29111 | GPL570 | whole blood | Normal (n = 3) | 25 samples |
|  | GSE48060 | GPL570 | Peripheral blood | Normal (n = 16) |  |
|  | GSE97320 | GPL570 | peripheral blood | Normal (n = 6) |  |
