# Supplementary material for: Hypoxia-associated genes predicting future risk of myocardial infarction: a GEO database-based study
Source: Front Cardiovasc Med. 2023 Jul 3;10:1068782. doi: 10.3389/fcvm.2023.1068782 (PMC10351911; doi:10.3389/fcvm.2023.1068782)
Supplement: Supplementary file 2 [file Table2.docx]

**Table S2：Clinical information of patients in training and test sets**.

| **Symbol** | **Database** | **Sample_source_name** | **Platform** | **Sex** | **Age** | **Time_point** | **Disease** | **Country** | **RNAdeg** | **batch** | **Type** |
| --- | --- | --- | --- | --- | --- | --- | --- | --- | --- | --- | --- |
| GSM721023 | GSE29111 | Sample_source_name | GPL570 | F | 65 | day_30 | UA | UK | 3.803028 | batch | train |
| GSM721022 | GSE29111 | Sample_source_name | GPL570 | M | 71 | day_30 | UA | UK | 4.137505 | batch | train |
| GSM721021 | GSE29111 | Sample_source_name | GPL570 | M | 48 | day_30 | UA | UK | 4.095107 | batch | train |
| GSM721020 | GSE29111 | Sample_source_name | GPL570 | M | 62 | day_30 | UA | UK | 4.044037 | batch | train |
| GSM721019 | GSE29111 | Sample_source_name | GPL570 | F | 56 | day_30 | UA | UK | 4.053124 | batch | train |
| GSM721018 | GSE29111 | Sample_source_name | GPL570 | M | 65 | day_30 | UA | UK | 3.420882 | batch | train |
| GSM721017 | GSE29111 | Sample_source_name | GPL570 | M | 74 | day_30 | UA | UK | 3.969281 | batch | train |
| GSM721016 | GSE29111 | Sample_source_name | GPL570 | M | 64 | day_30 | UA | UK | 3.729108 | batch | train |
| GSM721015 | GSE29111 | Sample_source_name | GPL570 | F | 65 | day_7 | UA | UK | 3.838356 | batch | train |
| GSM721014 | GSE29111 | Sample_source_name | GPL570 | M | 71 | day_7 | UA | UK | 4.015282 | batch | train |
| GSM721013 | GSE29111 | Sample_source_name | GPL570 | M | 48 | day_7 | UA | UK | 3.736921 | batch | train |
| GSM721012 | GSE29111 | Sample_source_name | GPL570 | M | 62 | day_7 | UA | UK | 3.757329 | batch | train |
| GSM721011 | GSE29111 | Sample_source_name | GPL570 | F | 56 | day_7 | UA | UK | 4.284585 | batch | train |
| GSM721010 | GSE29111 | Sample_source_name | GPL570 | M | 65 | day_7 | UA | UK | 3.617394 | batch | train |
| GSM721009 | GSE29111 | Sample_source_name | GPL570 | M | 74 | day_7 | UA | UK | 4.100999 | batch | train |
| GSM721008 | GSE29111 | Sample_source_name | GPL570 | M | 64 | day_7 | UA | UK | 3.332827 | batch | train |
| GSM721007 | GSE29111 | Sample_source_name | GPL570 | M | 41 | day_30 | MI | UK | 3.825357 | batch | train |
| GSM721006 | GSE29111 | Sample_source_name | GPL570 | M | 51 | day_30 | MI | UK | 4.115665 | batch | train |
| GSM721005 | GSE29111 | Sample_source_name | GPL570 | M | 57 | day_30 | MI | UK | 4.152345 | batch | train |
| GSM721004 | GSE29111 | Sample_source_name | GPL570 | M | 69 | day_30 | MI | UK | 3.96778 | batch | train |
| GSM721003 | GSE29111 | Sample_source_name | GPL570 | M | 64 | day_30 | MI | UK | 4.218683 | batch | train |
| GSM721002 | GSE29111 | Sample_source_name | GPL570 | M | 53 | day_30 | MI | UK | 4.396606 | batch | train |
| GSM721001 | GSE29111 | Sample_source_name | GPL570 | M | 39 | day_30 | MI | UK | 3.596103 | batch | train |
| GSM721000 | GSE29111 | Sample_source_name | GPL570 | F | 62 | day_30 | MI | UK | 3.944247 | batch | train |
| GSM720999 | GSE29111 | Sample_source_name | GPL570 | M | 41 | day_30 | MI | UK | 4.266739 | batch | train |
| GSM720998 | GSE29111 | Sample_source_name | GPL570 | M | 54 | day_30 | MI | UK | 3.695462 | batch | train |
| GSM720997 | GSE29111 | Sample_source_name | GPL570 | M | 66 | day_30 | MI | UK | 3.72888 | batch | train |
| GSM720996 | GSE29111 | Sample_source_name | GPL570 | M | 63 | day_30 | MI | UK | 4.030815 | batch | train |
| GSM720995 | GSE29111 | Sample_source_name | GPL570 | M | 54 | day_30 | MI | UK | 3.926364 | batch | train |
| GSM720994 | GSE29111 | Sample_source_name | GPL570 | F | 66 | day_30 | MI | UK | 4.453976 | batch | train |
| GSM720993 | GSE29111 | Sample_source_name | GPL570 | M | 50 | day_30 | MI | UK | 3.978589 | batch | train |
| GSM720992 | GSE29111 | Sample_source_name | GPL570 | M | 62 | day_30 | MI | UK | 3.821436 | batch | train |
| GSM720991 | GSE29111 | Sample_source_name | GPL570 | F | 67 | day_30 | MI | UK | 4.318508 | batch | train |
| GSM720990 | GSE29111 | Sample_source_name | GPL570 | M | 56 | day_30 | MI | UK | 3.88716 | batch | train |
| GSM720989 | GSE29111 | Sample_source_name | GPL570 | M | 41 | day_7 | MI | UK | 3.35743 | batch | train |
| GSM720988 | GSE29111 | Sample_source_name | GPL570 | M | 51 | day_7 | MI | UK | 4.017717 | batch | train |
| GSM720987 | GSE29111 | Sample_source_name | GPL570 | M | 57 | day_7 | MI | UK | 3.954667 | batch | train |
| GSM720986 | GSE29111 | Sample_source_name | GPL570 | M | 69 | day_7 | MI | UK | 3.883844 | batch | train |
| GSM720985 | GSE29111 | Sample_source_name | GPL570 | M | 64 | day_7 | MI | UK | 3.778695 | batch | train |
| GSM720984 | GSE29111 | Sample_source_name | GPL570 | M | 53 | day_7 | MI | UK | 4.555156 | batch | train |
| GSM720983 | GSE29111 | Sample_source_name | GPL570 | M | 39 | day_7 | MI | UK | 3.757196 | batch | train |
| GSM720982 | GSE29111 | Sample_source_name | GPL570 | F | 62 | day_7 | MI | UK | 3.914358 | batch | train |
| GSM720981 | GSE29111 | Sample_source_name | GPL570 | M | 41 | day_7 | MI | UK | 4.005461 | batch | train |
| GSM720980 | GSE29111 | Sample_source_name | GPL570 | M | 54 | day_7 | MI | UK | 3.409199 | batch | train |
| GSM720979 | GSE29111 | Sample_source_name | GPL570 | M | 66 | day_7 | MI | UK | 3.76714 | batch | train |
| GSM720978 | GSE29111 | Sample_source_name | GPL570 | M | 63 | day_7 | MI | UK | 4.066636 | batch | train |
| GSM720977 | GSE29111 | Sample_source_name | GPL570 | M | 54 | day_7 | MI | UK | 3.687232 | batch | train |
| GSM720976 | GSE29111 | Sample_source_name | GPL570 | F | 66 | day_7 | MI | UK | 4.59983 | batch | train |
| GSM720975 | GSE29111 | Sample_source_name | GPL570 | M | 50 | day_7 | MI | UK | 3.840002 | batch | train |
| GSM720974 | GSE29111 | Sample_source_name | GPL570 | M | 62 | day_7 | MI | UK | 3.852873 | batch | train |
| GSM720973 | GSE29111 | Sample_source_name | GPL570 | F | 67 | day_7 | MI | UK | 4.005282 | batch | train |
| GSM720972 | GSE29111 | Sample_source_name | GPL570 | M | 56 | day_7 | MI | UK | 3.956413 | batch | train |
| GSM855107 | GSE34781 | Sample_source_name | GPL570 | NA | NA | NA | UA | China | 3.094154 | batch | train |
| GSM855106 | GSE34781 | Sample_source_name | GPL570 | NA | NA | NA | UA | China | 3.151219 | batch | train |
| GSM1167072 | GSE48060 | Sample_source_name | GPL570 | NA | NA | NA | MI | USA | 4.165676 | batch | train |
| GSM1167073 | GSE48060 | Sample_source_name | GPL570 | NA | NA | NA | MI | USA | 3.875168 | batch | train |
| GSM1167074 | GSE48060 | Sample_source_name | GPL570 | NA | NA | NA | MI | USA | 4.794258 | batch | train |
| GSM1167075 | GSE48060 | Sample_source_name | GPL570 | NA | NA | NA | MI | USA | 4.256288 | batch | train |
| GSM1167076 | GSE48060 | Sample_source_name | GPL570 | NA | NA | NA | MI | USA | 4.689691 | batch | train |
| GSM1167077 | GSE48060 | Sample_source_name | GPL570 | NA | NA | NA | MI | USA | 4.684582 | batch | train |
| GSM1167078 | GSE48060 | Sample_source_name | GPL570 | NA | NA | NA | MI | USA | 4.252259 | batch | train |
| GSM1167079 | GSE48060 | Sample_source_name | GPL570 | NA | NA | NA | MI | USA | 4.415026 | batch | train |
| GSM1167080 | GSE48060 | Sample_source_name | GPL570 | NA | NA | NA | MI | USA | 4.577792 | batch | train |
| GSM1167081 | GSE48060 | Sample_source_name | GPL570 | NA | NA | NA | MI | USA | 4.886332 | batch | train |
| GSM1167082 | GSE48060 | Sample_source_name | GPL570 | NA | NA | NA | MI | USA | 3.945738 | batch | train |
| GSM1167083 | GSE48060 | Sample_source_name | GPL570 | NA | NA | NA | MI | USA | 4.390982 | batch | train |
| GSM1167084 | GSE48060 | Sample_source_name | GPL570 | NA | NA | NA | MI | USA | 3.830856 | batch | train |
| GSM1167085 | GSE48060 | Sample_source_name | GPL570 | NA | NA | NA | MI | USA | 4.629541 | batch | train |
| GSM1167086 | GSE48060 | Sample_source_name | GPL570 | NA | NA | NA | MI | USA | 3.927685 | batch | train |
| GSM1167087 | GSE48060 | Sample_source_name | GPL570 | NA | NA | NA | MI | USA | 4.135902 | batch | train |
| GSM1167088 | GSE48060 | Sample_source_name | GPL570 | NA | NA | NA | MI | USA | 4.018437 | batch | train |
| GSM1167089 | GSE48060 | Sample_source_name | GPL570 | NA | NA | NA | MI | USA | 4.578666 | batch | train |
| GSM1167090 | GSE48060 | Sample_source_name | GPL570 | NA | NA | NA | MI | USA | 4.288477 | batch | train |
| GSM1167091 | GSE48060 | Sample_source_name | GPL570 | NA | NA | NA | MI | USA | 4.246164 | batch | train |
| GSM1167092 | GSE48060 | Sample_source_name | GPL570 | NA | NA | NA | MI | USA | 4.579207 | batch | train |
| GSM1167093 | GSE48060 | Sample_source_name | GPL570 | NA | NA | NA | MI | USA | 4.440343 | batch | train |
| GSM1167094 | GSE48060 | Sample_source_name | GPL570 | NA | NA | NA | MI | USA | 4.343675 | batch | train |
| GSM1167095 | GSE48060 | Sample_source_name | GPL570 | NA | NA | NA | MI | USA | 4.509716 | batch | train |
| GSM1167096 | GSE48060 | Sample_source_name | GPL570 | NA | NA | NA | MI | USA | 5.011606 | batch | train |
| GSM1167097 | GSE48060 | Sample_source_name | GPL570 | NA | NA | NA | MI | USA | 3.897084 | batch | train |
| GSM1167098 | GSE48060 | Sample_source_name | GPL570 | NA | NA | NA | MI | USA | 4.668016 | batch | train |
| GSM1167099 | GSE48060 | Sample_source_name | GPL570 | NA | NA | NA | MI | USA | 4.508455 | batch | train |
| GSM1167100 | GSE48060 | Sample_source_name | GPL570 | NA | NA | NA | MI | USA | 4.359656 | batch | train |
| GSM1167101 | GSE48060 | Sample_source_name | GPL570 | NA | NA | NA | MI | USA | 4.011482 | batch | train |
| GSM1167122 | GSE48060 | Sample_source_name | GPL570 | NA | NA | NA | MI | USA | 4.194033 | batch | train |
| GSM2561551 | GSE97320 | Sample_source_name | GPL570 | NA | 65 | NA | MI | China | 4.022767 | batch | train |
| GSM1498376 | GSE61144 | Sample_source_name | GPL6106 | NA | NA | NA | MI | Korea | NA | batch | test |
| GSM1498377 | GSE61144 | Sample_source_name | GPL6106 | NA | NA | NA | MI | Korea | NA | batch | test |
| GSM1498378 | GSE61144 | Sample_source_name | GPL6106 | NA | NA | NA | MI | Korea | NA | batch | test |
| GSM1498379 | GSE61144 | Sample_source_name | GPL6106 | NA | NA | NA | MI | Korea | NA | batch | test |
| GSM1498380 | GSE61144 | Sample_source_name | GPL6106 | NA | NA | NA | MI | Korea | NA | batch | test |
| GSM1498381 | GSE61144 | Sample_source_name | GPL6106 | NA | NA | NA | MI | Korea | NA | batch | test |
| GSM1498382 | GSE61144 | Sample_source_name | GPL6106 | NA | NA | NA | MI | Korea | NA | batch | test |
