# Supplementary material for: Hypoxia-associated genes predicting future risk of myocardial infarction: a GEO database-based study
Source: Front Cardiovasc Med. 2023 Jul 3;10:1068782. doi: 10.3389/fcvm.2023.1068782 (PMC10351911; doi:10.3389/fcvm.2023.1068782)

**Figure S1: GEO Data De-Batch Processing.** Intensity (A) before correction and (B) after correction in all samples of GPL570 (including GSE29111, GSE34781, GSE48060, and GSE97320). (C) Remove samples with low connectivity. PCA analysis of (D) pre-calibrated and (E) post-calibrated samples from the GPL 570 platform. Calibrated samples from GPL 6106 platform (F). PCA analysis of training set samples (GPL 570+GPL 6106) (G) before and (H) after correction. (I) Intensity corrected for testing set samples (GSE60993).


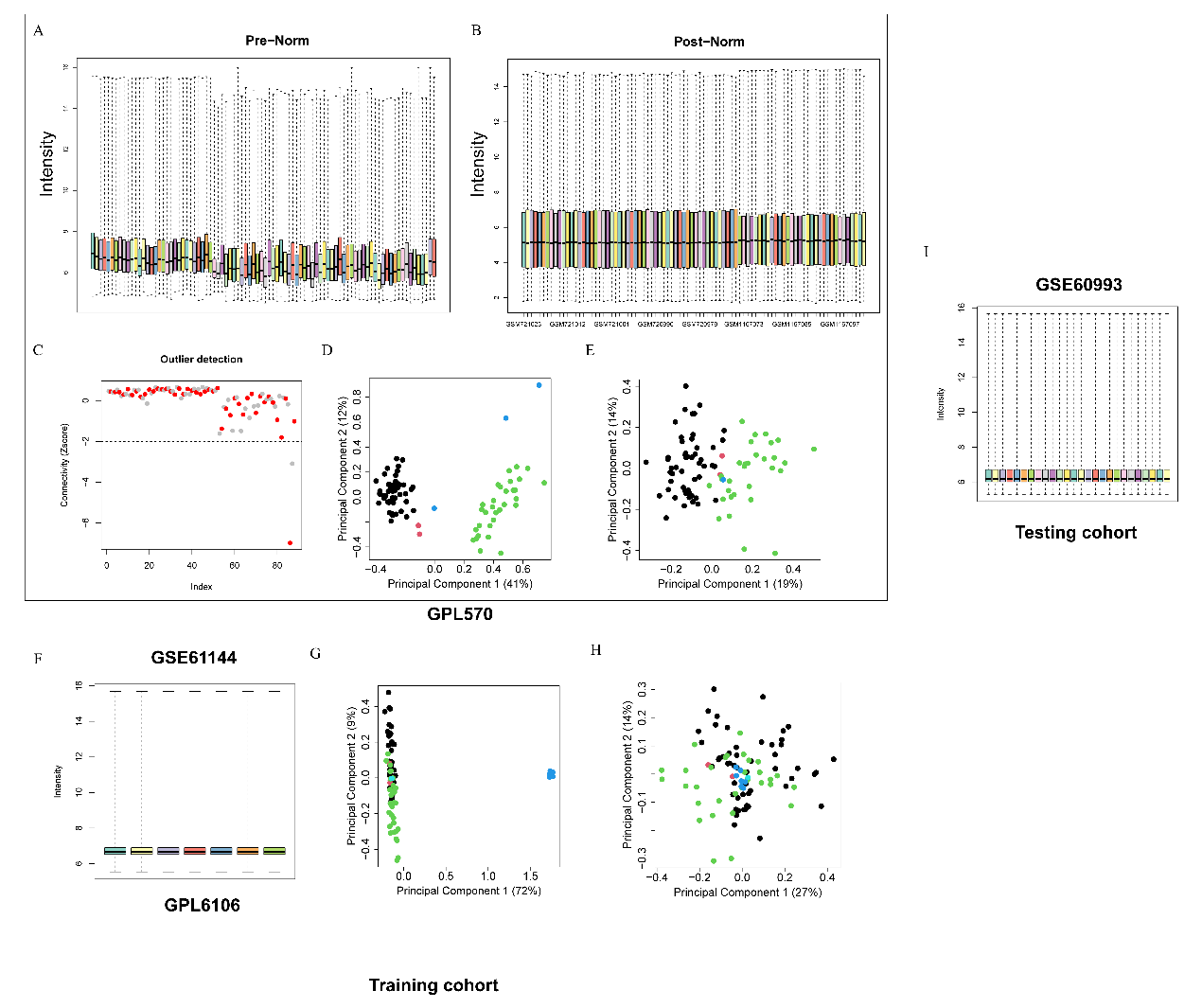


**Figure S2: Relationship of riskscore and clinical information including (A) age and (B) gender.**

**
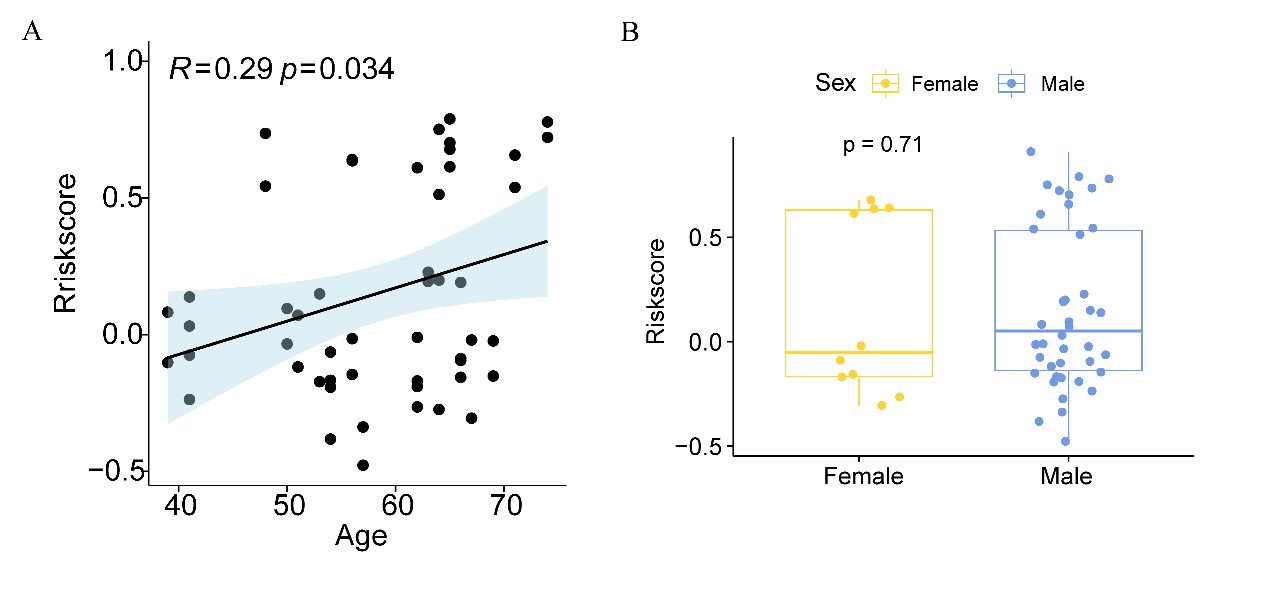
**

**Figure S3: ROC curve of a single lncRNA.** The top 8 best ROC curves of a single lncRNA in the (A) training set and (B) test set.


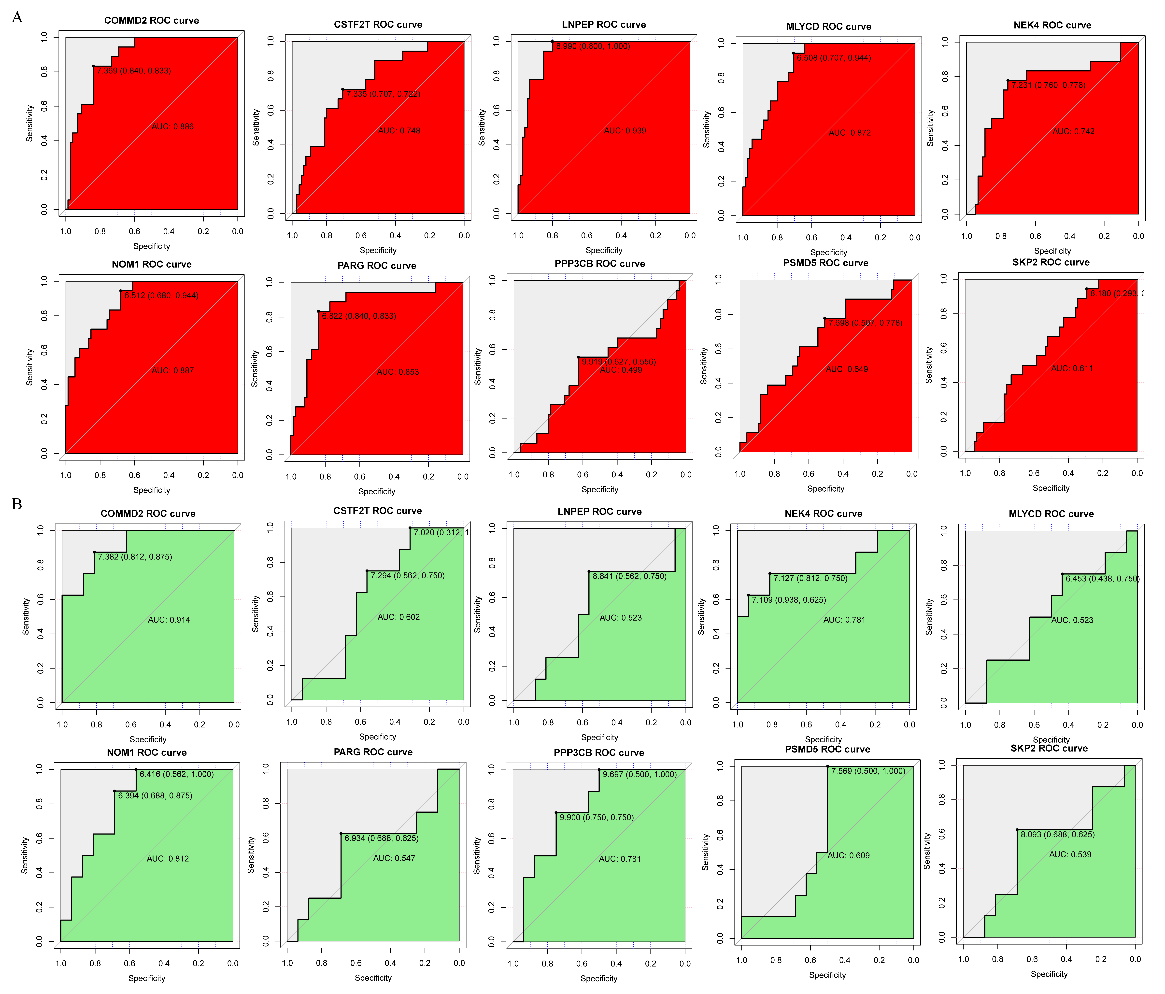


**Figure S4: Correlation analysis of risk score and immune cells.**


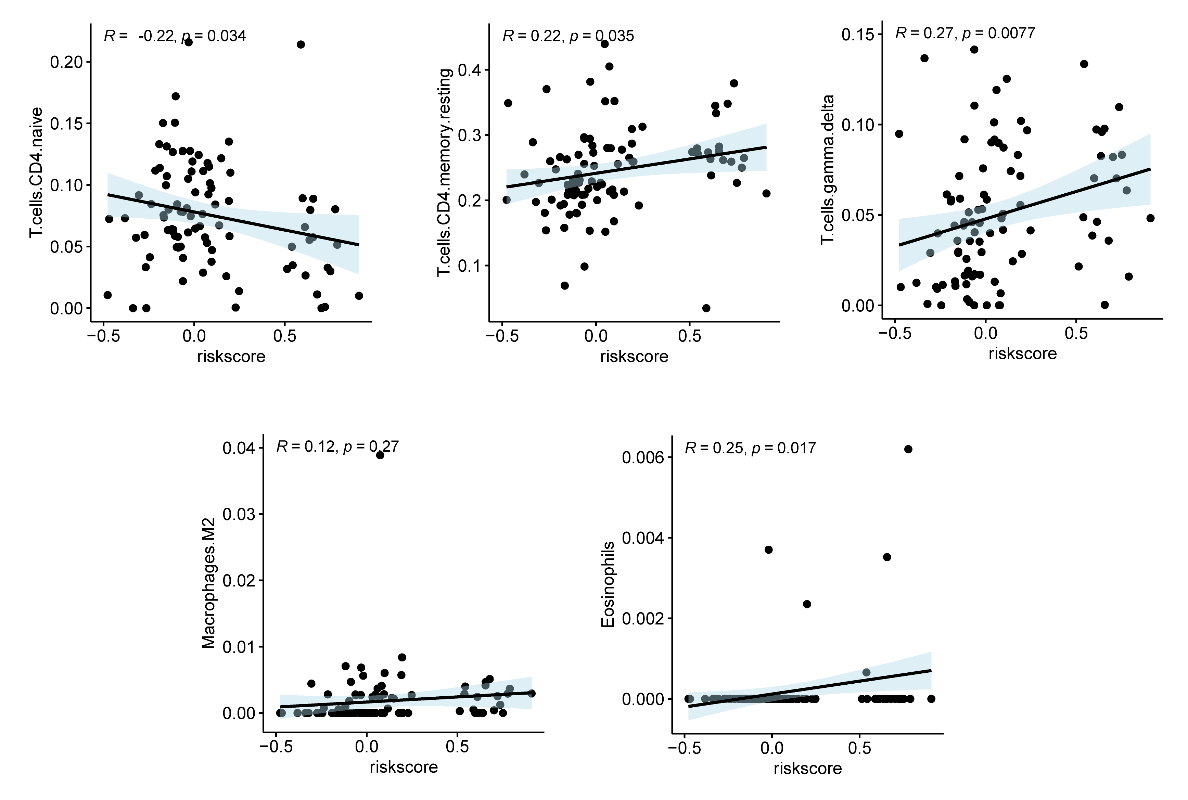

Supplement: Supplementary file 4 [file Datasheet1.docx]
